# Early nasogastric feeding versus parenteral nutrition in severe acute pancreatitis: A retrospective study

**DOI:** 10.12669/pjms.326.11278

**Published:** 2016

**Authors:** Yulong Tao, Chengwu Tang, Wenming Feng, Ying Bao, Hongbin Yu

**Affiliations:** 1 Dr. Yulong Tao, MD, Department of General Surgery, First People’s Hospital affiliated to Huzhou University Medical College, Huzhou, Zhejiang Province, China; 2 Dr. Chengwu Tang, MD, Department of General Surgery, First People’s Hospital affiliated to Huzhou University Medical College, Huzhou, Zhejiang Province, China; 3 Dr. Wenming Feng, MD, Department of General Surgery, First People’s Hospital affiliated to Huzhou University Medical College, Huzhou, Zhejiang Province, China; 4 Dr. Ying Bao, MD, Department of General Surgery, First People’s Hospital affiliated to Huzhou University Medical College, Huzhou, Zhejiang Province, China; 5 Dr. Hongbin Yu, MD, Department of General Surgery, First People’s Hospital affiliated to Huzhou University Medical College, Huzhou, Zhejiang Province, China

**Keywords:** Enteral Nutrition, Severe Acute Pancreatitis, Total Parenteral Nutrition

## Abstract

**Objective:**

To compare the efficacy and safety of early nasogastric enteral nutrition (EN) with total parenteral nutrition (TPN) in patients with severe acute pancreatitis (SAP).

**Methods:**

From July 2008 to July 2014,185 patients with SAP admitted to our centre were enrolled in this retrospective study. They were divided into EN group (n=89) and TPN group (n=96) based on the nutrition support modes. Patients in EN group received nasogastric EN support, while patients in TPN group received TPN support within 72 hours of disease onset. The medical records were reviewed and clinical factors were retrospectively analyzed.

**Results:**

There were no significant differences in baseline characteristics between two groups. EN group had significantly lower incidence of pancreatic infections (P=0.0333) and extrapancreatic infections (P=0.0431). Significantly shorter hospital stay (P=0.0355) and intensive-care stay (P=0.0313) were found in EN group. TPN group was found to have significantly greater incidence of multiple organ dysfunction syndrome (MODS) (P=0.0338) and mortality (P=0.0382). Moreover, the incidence of hyperglycemia was significantly higher in TPN group (P=0.0454).

**Conclusions:**

Early nasogastric EN was feasible and significantly decreased the incidence of infectious complications as well as the frequency of MODS and mortality caused by SAP.

## INTRODUCTION

Severe acute pancreatitis (SAP) is a common cause of emergency hospital admission,^1^ with an increase in the incidence during the past 30 years, which can induce vascular leakage, shock, systemic inflammatory response syndrome, organ dysfunctions and even mortality.^2^,^3^ Nutritional support should be an integral part of patient care and started early in the course of disease.^4^,^5^ Theoretically, enteral nutrition (EN) should minimize the incidence of infectious complications in patients with SAP, as it probably reduces bacterial translocation and the consequent sepsis by enhancing intestinal barrier function.^6^ However, another clinical randomized study found that early nasogastric EN resulted in a higher overall early complication rate than total parenteral nutrition (TPN) with no beneficial effects on intestinal permeability or the inflammatory response.^7^ On the basis of aforementioned facts, we conducted this retrospective study to evaluate the efficacy and safety of early nasogastric enteral nutrition as compared with TPN in patients with SAP.

## METHODS

### Patients

Between June 2008 and June 2014, 185 patients admitted to First People’s Hospital affiliated to Huzhou University Medical College (Zhejiang Province, China) with a clinical diagnosis of SAP within 48h of disease onset were enrolled in this retrospective study. According to the nutrition support modes, they were divided into two groups: 96 in TPN group and 89 in EN group.

The diagnostic and classification criteria for SAP included at least two of the following features^8^:

1. Abdominal pain consistent with AP;
2. Amylase activity at least four folds of the upper limit of normal; and
3. Characteristic findings of AP on contrast-enhanced computed tomography (CT) and, less commonly, on magnetic resonance imaging or transabdominal ultrasonography.

The exclusion criteria included the following:

1. Previously with cardiac failure or pulmonary edema;
2. Pregnant;
3. Underwent early surgical treatment;
4. Known allergy to any of the ingredients;
5. With poorly controlled diabetes

This study was conducted in accordance with the principles of the Declaration of Helsinki and “Good Clinical Practice” guidelines. Written informed consent was obtained from all patients and study protocol was approved by the Institutional Review Board.

### Treatment

Patients were monitored and treated according to clinical routine including pain control, symptomatic and organ supportive treatment. Broad-spectrum antibiotic therapy was used before microbiological examination.^9^ Intervention was indicated when infection of pancreatic necrosis was proven by the fine needle aspiration or when sepsis persisted despite maximal support on the intensive care unit. Patients with infected pancreatic necrosis were operated upon (necrosectomy and open packing or closed continuous lavage). Patients with pancreatic abscess were an indication for percutaneous drainage.

Nutrition support was begun within 72 hours of disease onset for a minimum of seven days in both groups, supplying daily 30 kcal/kg and 1.5 g/kg of protein, based on admission weight. In both groups, standard formulas without specific immunomodulating nutrients were used. TPN (Kabiven PI, Sino-Swed LTD., Wuxi, China) was delivered through a central venous catheter, initially infused at half of the calculated energy requirements; then increased over 48 hours to achieve 100% of the target energy rate. EN (Fresubin original, Sino-Swed LTD.) was administrated through a nasogastric feeding tube (size 8FG Flocare polyurethane feeding tubes, Nutricia LTD., Trowbridge, UK), at a rate of 25 ml/hour and increased by 10 ml/hour every 6 hours, until the desired caloric intake was reached. The NG feeding tubes were inserted on the ward by the medical staff. The position of the feeding tube was checked by aspiration and pH measurement. A chest radiograph was performed if necessary. The aim was to reach full nutrition within 48 hours after initiation. Oral feeding was reintroduced when amylase and CRP levels had decreased and abdominal pain had resolved. Regular hospital diet was introduced gradually, in general initially starting with liquid and then solid food.

### Outcome Measurements

The medical documents were reviewed for data collection. The incidence of pancreatic infectious complications (i.e. infected pancreatic necrosis and pancreatic abscess), extrapancreatic infectious complications, feeding-associated complications (diarrhea, abdominal bloating, hyperglycemia, dislodged nasogastric tubes, etc.) and organ failure during hospital admission were retrospectively compared. Diagnosis of pancreatic infectious complications was based on microbiological examination (Gram stain and culture for aerobic and anaerobic germs and fungi) of samples obtained by ultrasound-guided fine-needle aspiration of (peri-) pancreatic collections and confirmed in pancreatic tissue specimens obtained either during surgery or autopsy in patients operated upon or who died, respectively.^10^

Also, nutrition support outcomes, total hospital, intensive-care stay and mortality rate were compared. Hyperglycemia during feeding was defined as blood glucose >10 mmol/L. Local and systemic complications of acute pancreatitis were defined as previously reported.^11^ To assess organ failure, the Marshall score was used.^12^

### Statistical Analysis

All measurements were expressed as mean ± SD. The statistical analyses were performed using the two-sample *t*-test and adjusted Chi-square test for the two groups. The Exact *Chi-square* test was also used if individual cell size was less than five counts. *P* value <0.05 was considered statistically significant.

## RESULTS

### Baseline Characteristics of Patients

No significant difference was found in terms of gender, age, BMI, body weight, underlying comorbidity, etiological factor, APACHE II score and CRP level at admission (Table-I).

**Table-I:** Baseline characteristics of patients.

	TPN group (n=96)	EN group (n=89)	P value
Gender			0.9649
Male	72	67
Female	24	22
*Underlying Comorbidity*			
Hypertension	11	12	0.6775
Coronary heart disease	4	2	0.7482
Diabetes mellitus	10	8	0.7440
Fatty liver	12	13	0.6762
Chronic obstructive pulmonary disease	4	5	0.9073
Etiological Factor			0.9145
Biliary tract stone	54	50	
Hyperlipidemia	12	14	
Alcohol intake	26	22	
Others	4	3	
Age (year)	61.3±6.1	62.9±6.5	0.0858
BMI (kg/m^2^)	22.6±3.1	23.3±3.4	0.1447
Body weight (kg)	77.7±9.5	76.3±8.4	0.2912
APACHE II Score	11.6±2.4	12.1±2.7	0.1841
CRP level (mg/L)	285.4±44.7	295.5±47.6	0.1384
Admission (hours from disease onset)	34.6±4.5	35.4±5.2	0.2637

### Pancreatic and Extrapancreatic Infectious Complications

The incidence of pancreatic infections was significantly lower in EN group (12/89 vs.25/96, P=0.0333). (Table-II). Also, the incidence of extrapancreatic infections was significantly lower in EN group (14/89 vs.27/96, P=0.0431) (Table-II). Five patients (two in the TPN group and three in EN group) had concomitant extrapancreatic infections.

**Table-II:** Complications and Organ Failures.

	TPN group (n=96)	EN group (n=89)	P value
Pancreatic Infection	25	12	0.0333
Infected pancreatic necrosis	11	5	0.1591
Pancreatic abscess	14	7	0.1512
Extrapancreatic Infection	27	14	0.0431
Urinary infection	7	4	0.4227
Pneumonia	14	13	0.9964
Infections of the central venous catheter	8	-	-
Feeding-associated Complications	34	33	0.8147
Dislodged nasogastric tubes	-	5	-
Diarrhea	3	9	0.0545
Abdominal bloating	-	8	-
Hyperglycemia	32	18	0.0454
Single Organ Failure	36	26	0.1749
Acute renal failure	11	8	0.5814
Acute respiratory failure	13	11	0.8115
Shock	4	2	0.7482
Coagulopathy	8	5	0.6642
Multiple Organ Dysfunction Syndrome	13	4	0.0338

### Feeding-Associated Complications

Total feeding-associated complications did not statistically differ between two groups (34/96 vs. 33/89, P=0.8147) (Table-II). Despite our efforts, tubes were displaced in five cases and a replacing maneuver was required. Eight patients in EN group developed abdominal bloating. In these patients, the volume of their enteral nutrition was temporarily reduced for between one and two days. A troublesome diarrhea occurred in nine patients in EN group. In these patients, the rate of feeding was temporarily reduced and four of the patients received loperamide as well. In all patients resumption of full enteral support was achieved for the remainder of the feeding period. In TPN group, three patient developed diarrhea. The incidence of hyperglycemia during feeding was significantly higher in TPN group (32/96 vs. 18/89, P = 0.0454) (Table-II). Insulin therapy was initiated once hyperglycemia was found.

### Organ Failures and Mortality

In TPN group, 36 of 96 patients developed single organ failure, whereas 26 of 89 patients in EN group had single organ failure (P=0.1749). However, incidence of multiple organ dysfunction syndrome (MODS) was significantly higher in TPN group (13/96 vs. 4/89, P=0.0338) (Table-II).

A total of 14 patients died in two groups. The mortality rate was significantly greater in TPN group (11/96 vs. 3/89, P=0.0382) (Table-III). Three patients in TPN group and two patients in EN group died in first week of illness, four patients in TPN group and one patient in EN group died in second week of uncontrollable MODS, and four patients in TPN group died in third week after disease on set of multiple organ systems failure resulting from infected pancreatic necrosis (Table-III).

**Table-III:** Clinical Outcomes.

	TPN group (n=96)	EN group (n=89)	P value
Nutrition Support Outcomes			
Initiation (hours after disease onset)	61.4±6.4	62.3±7.1	0.3658
Interval to full rate (hours after initiation)	32.2±5.4	33.4±5.9	0.1503
Completely discontinued (days after initiation)	9.2±1.4	9.6±1.6	0.0839^a^
Total Hospital Stay (day)	22.4±4.3	21.1±3.7	0.0355^a^
Intensive-care Stay (day)	8.3±2.3	7.6±1.9	0.0313^a^
Mortality (case)	11	3	0.0382

### Clinical Outcomes

The nutrition support was initiated in 61.4±6.4 hours after disease onset in TPN group and 62.3±7.1 hours in EN group (P=0.3658) (Table-III). Full rate of nutrition was achieved in all patients and there was no significant difference in the time to full rate of nutrition support after initiation (TPN: 32.2±5.4 hours vs. EN: 33.4±5.9 hours, P=0.1503). Intake of liquid or solid food without nutrition supplement was achieved in 9.2±1.4 days in TPN group and 9.6±1.6 days in EN group (P=0.0715) (Table-III). No patient interrupted their oral feeding because of pain relapse. Nutrition support was maintained until death in dead cases.

The length of hospital stay was significantly shorter in EN group (21.1±3.7 days vs. 22.4±4.3 days, P=0.0355). Likewise, intensive-care stay in EN group was significantly shorter than in TPN group (7.6±1.9 days vs.8.3±2.3 days, P=0.0313) (Table-III).

## DISCUSSION

Mortality of SAP has two peaks, i.e., “early” during the first week and “late” after 1 to 3 weeks.^13^ The second peak in mortality usually involves MODS together with infections, which are frequently caused by gram-negative bacteria. The predominance of gram-negative bacteria isolated from infected pancreatic tissue resemble common gastrointestinal flora, suggesting that they reach the pancreas by translocation from the gut. Thereby, preservation or restoration of the intestinal barrier function may have a beneficial impact on infectious morbidity from severe acute pancreatitis and may reduce mortality. Early enteral feeding is recommended to preserve the intestinal barrier to prevent bacterial translocation along with avoiding the complications associated with parenteral nutrition in SAP.^14^ Proper timing is probably crucial for achieving success with therapeutic interventions, including modulation of inflammatory mediator production and release. In SAP, a therapeutic window exists up to about 48 and 72 hours from pain onset, i.e., in the time phase usually required for the development of remote organ dysfunction.^15^ In present study, we initialed TPN and EN in 61.4±6.4 hours and 62.3±7.1 hours after disease onset respectively (P=0.3658). In present study, mortality rate of EN group was significant lower. In some reports, the majority of deaths in SAP are attributed to infected necrosis and secondary multi-organ failure, which means late mortality.^16^-^18^ Notably, six patients in TPN group and none in EN group died in the second week after disease onset. This would suggest that early enteral feeding could efficiently preserve the intestinal barrier to prevent bacterial translocation and reduce ‘late’ mortality in patients with SAP.

Pancreatic infections and subsequent septic complications have emerged as the main risk factor for late death in severe acute pancreatitis. It is widely accepted that early enteral feeding is critical to improving outcomes in patients with SAP since the first demonstrated the beneficial effect of an elemental enteral diet in 6 patients with acute pancreatitis in 1973.^14^ We found that more patients developed pancreatic infectious complications (P=0.0333) and extrapancreatic infections (P=0.0431) in TPN group. A significant decrease in the incidence of infectious complications in EN group may lead to the reduction of surgical interventions in the enter ally fed patients. Consequently, the length of hospital stay and intensive-care stay in EN group was significantly shorter. Similar to that mentioned above regarding the incidence of infectious complications, the incidence of MODS (P=0.0338) was significantly lower in EN group.

In present study, full rate of nutrition was achieved in all patients and there was no significant difference in the time to full rate of nutrition support after initiation. Total feeding-associated complications did not statistically difference between two groups (P=0.8147). Hyperglycemia is common in SAP, and in the present study incidence of hyperglycemia during feeding was significantly lower in EN group (P=0.0454). The effects of normoglycemia in SAP have not yet been studied, but potentially this concept might further improve outcome also in patients with SAP.

In conclusion, this retrospective study has confirmed that nasogastric EN may be safely delivered to patients with SAP without causing feeding-associated complications. Based on the improved outcomes, nasogastric EN should be started within 72 hours after disease onset in all patients with severe acute pancreatitis. Because of the retrospective nature and the small sample size, further prospective study with large sample size is needed to confirm the results of our study.
